# Angiotensin II induces cholesterol accumulation and injury in podocytes

**DOI:** 10.1038/s41598-017-09733-w

**Published:** 2017-09-06

**Authors:** Yingjie Yang, Qian Yang, Jian Yang, Yiqiong Ma, Guohua Ding

**Affiliations:** Division of Nephrology, Renmin Hospital of Wuhan University, Wuhan, Hubei 430060 China

## Abstract

Angiotensin II (Ang II) is a risk factor for the initiation and progression of chronic kidney disease (CKD), as elevated Ang II levels can lead to podocyte injury. However, there have been no studies on the role of Ang II in lipid metabolism or on podocyte injury caused by lipid dysfunction. Our study showed that Ang II induced lipid droplet (LD) accumulation and expression of the LD marker adipose differentiation-related protein (ADRP) in podocytes, and the extent of lipid deposition could be alleviated by losartan. Our study also demonstrated that Ang II increased the content of cholesterol in podocytes, which is an LD component, and this change was accompanied by decreased expression of the cholesterol efflux-related molecule ATP-binding cassette transporter-1 (ABCA1) and increased expression of the cholesterol uptake-related molecule LDL receptor (LDLR) and the cholesterol synthesis-related molecules sterol regulatory element-binding protein (SREBP1 and SREBP2) and 3-hydroxy-3-methylglutaryl CoA reductase (HMGCR). Pretreating podocytes with methyl-β-cyclodextrin (CD), which induces cholesterol efflux, decreased Ang II-mediated cholesterol accumulation and Ang II-induced podocyte apoptosis and maintained the podocyte cytoskeleton and spreading. These results suggested that Ang II induced podocyte cholesterol accumulation by regulating the expression of cholesterol metabolism-related molecules and that the subsequent cholesterol metabolism dysfunction resulted in podocyte injury.

## Introduction

Podocytes are visceral glomerular epithelial cells consisting of a cell body and major and minor foot processes. Foot processes from neighboring podocytes develop into a slit diaphragm (SD)^1^. The SD is a lipid raft-like structure rich in cholesterol that has important functions in the regulation of membrane fluidity, membrane protein trafficking, and the assembly of signaling molecules^2^. Podocyte-specific proteins, such as podocin, can bind and recruit cholesterol to contribute to SD formation^3^. These features highlight the importance of cholesterol homeostasis in podocytes and suggest that cholesterol serves as an important regulator in the development of proteinuria kidney diseases.

Cellular cholesterol homeostasis is regulated by *de novo* synthesis and cholesterol influx and efflux^3^. *De novo* synthesis is mainly controlled by the rate-limiting enzyme HMGCR, which can be regulated on a transcriptional level by SREBP1 and SREBP2, while cholesterol influx is mediated by LDLR, and cholesterol efflux is primarily mediated by ABCA1^3, 4^. Several studies have reported that substantial amounts of cholesterol are deposited in the glomeruli of diabetic kidney disease (DKD) patients, who exhibit decreased levels of cholesterol efflux^5–9^. Inducing cholesterol efflux with CD can alleviate podocyte injury *in vitro* and lead to reductions in albuminuria, mesangial expansion and cortical cholesterol content in ob/ob mice^5^. Furthermore, some researchers have reported lipid accumulation in the tubular epithelial and vascular wall cells of Ang II-treated rats^10^. However, no studies have evaluated the role of Ang II in podocyte lipid metabolism.

Ang II, a mediator of the renin-angiotensin system (RAS), is a notable risk factor for the initiation and progression of CKD, and elevated Ang II levels can lead to podocyte injury^11^. CD is a well-known cholesterol efflux inducer that has a strong affinity for the membrane surface and thus destabilizes the local packing of cholesterol in the plasma membrane to promote cholesterol extraction^12^. This characteristic is exploited by hydroxy-propyl-β-cyclodextrin (HPBCD), which has been approved by the U.S. Food and Drug Administration (FDA) for the treatment of Niemann-Pick disease^4^. In the present study, we hypothesized that Ang II would induce podocyte cholesterol accumulation and injury and that CD-induced cholesterol efflux would protect podocytes from Ang II-mediated damage.

## Results

### Effect of Ang II on LD formation in podocytes

**Figure 1:**
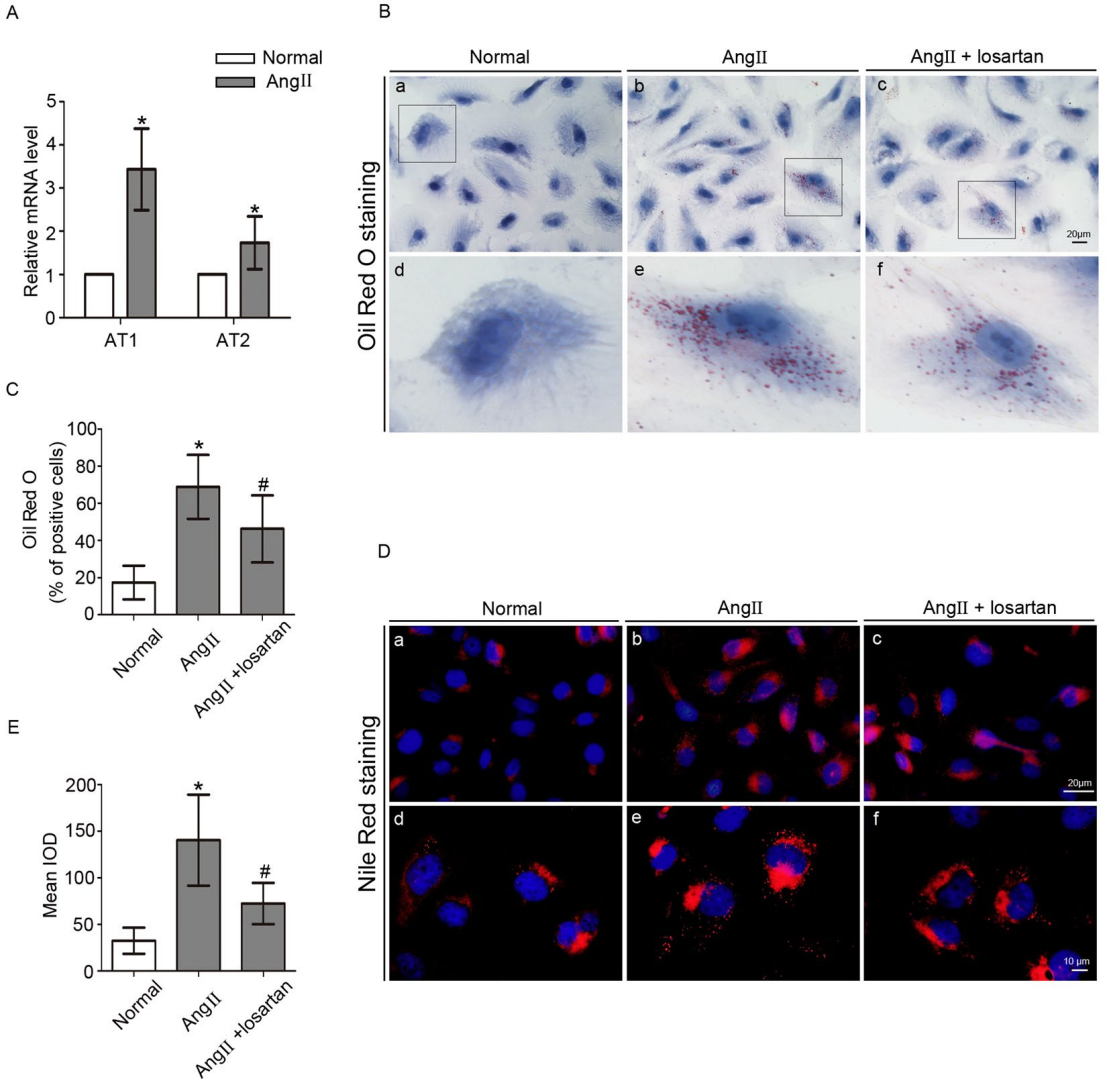
Effect of Ang II on LD formation in podocytes. Podocytes were stimulated with Ang II (10^−7^ M) for 24 h with or without losartan (10^−5^ M). (**A**) Quantitative analysis of the mRNA levels of AT1 and AT2 receptors. n = 3, *p < 0.05 vs. the normal group. (**B**) a–c, Representative Oil Red O staining images of podocytes in different groups (magnification ×400). Scale bar, 20 μm; d–f, Magnification of a–c. (**C**) Quantitative analysis of the number of Oil Red O positive cells in different groups. n = 10, *p < 0.05 vs. the normal group. ^#^ *p* < 0.05 vs. the Ang II group. (**D**) a–c, Representative images of Nile Red-stained podocytes in different groups (original magnification ×400). Scale bar, 20 μm; d–f, magnification ×1000, scale bar, 10 μm. (**E**) Quantitative analysis of the mean integrated optical density (IOD) of Nile Red staining in podocytes. n = 6, *p < 0.05 vs. the normal group. ^#^ *p* < 0.05 vs. the Ang II group.

Podocytes were exposed to Ang II (10^−7^ M) for 24 h. The mRNA levels of Ang II type 1 (AT1) and type 2 receptors (AT2) were increased by Ang II treatment (Fig. 1A). We used Oil Red O staining to evaluate the effect of Ang II on LD formation in podocytes, and found that in contrast to the minimal or absent staining in normal podocytes, many Ang II-treated podocytes were stained with Oil Red O. We next assessed the effect of the AT1 blocker losartan on LD formation. The data showed that the positive cell number and the degree of staining induced by Ang II were ameliorated by losartan (Fig. 1B and C). LD distribution in podocytes was also assessed by Nile Red staining, which showed the same phenomenon as that of Oil Red O (Fig. 1D and E). Thus, these findings indicated that Ang II promoted LD formation in podocytes.

### Effect of Ang II on ADRP expression in podocytes

**Figure 2:**
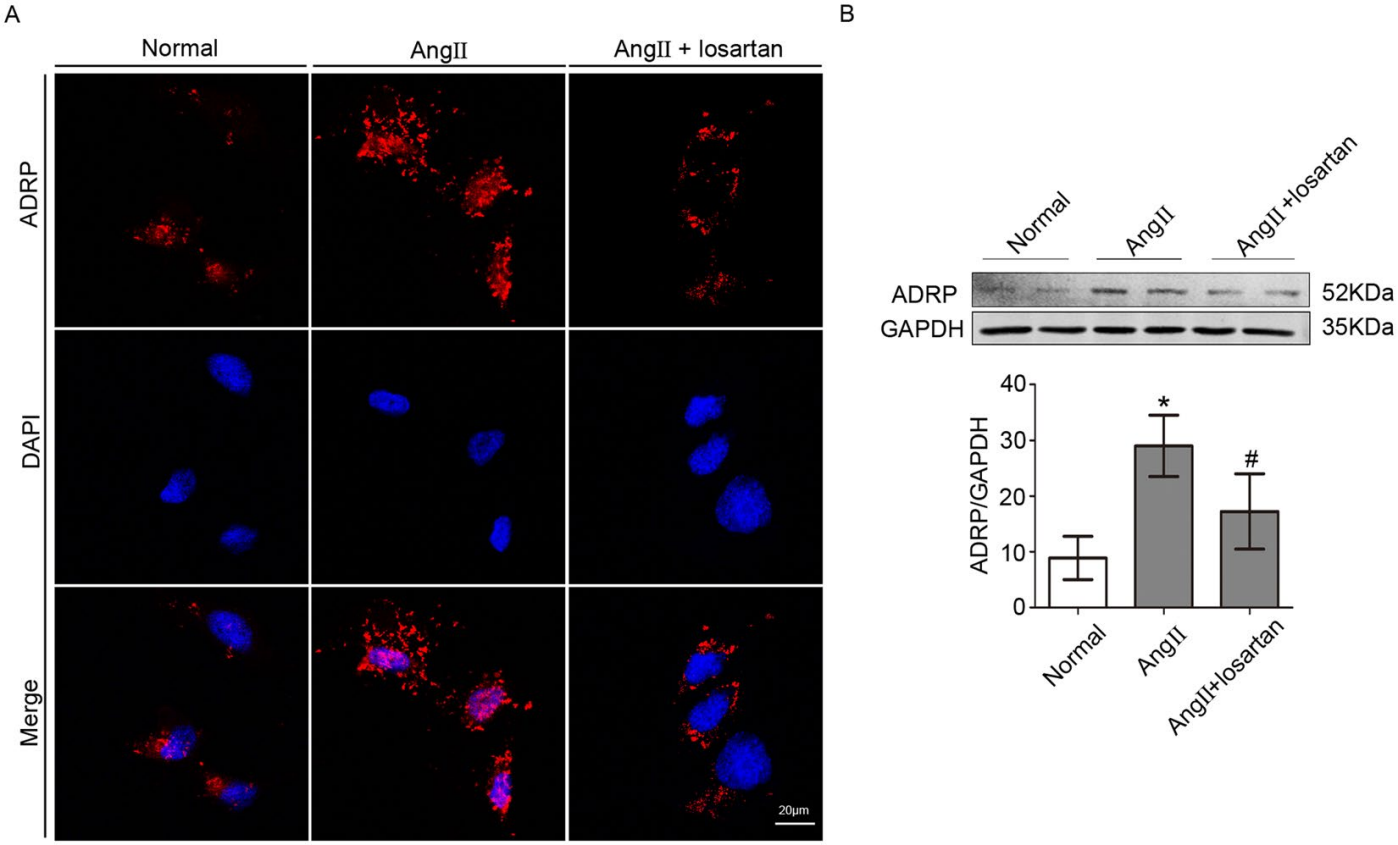
Effect of Ang II on ADRP expression in podocytes. (**A**) Representative immunofluorescence images of the LD marker ADRP. (magnification ×400). Scale bar, 20 μm. (**B**) Representative Western blot and quantitation of the ADRP expression in different groups. n = 3, *p < 0.05 vs. the normal group, ^#^ *p* < 0.05 vs. the Ang II group. For clarity lanes were cropped from the same gel. Full-length blots are presented in Fig. S3.

ADRP is an LD marker^8^. We next evaluated the effect of Ang II on ADRP expression using immunofluorescence microscopy. Low ADRP expression was found in normal podocytes, while high ADRP expression was observed in Ang II-treated podocytes, with punctate distribution in the cytoplasm. Then, we assessed the role of losartan in ADRP expression. We observed lower ADRP expression in podocytes treated with losartan compared with Ang II-treated cells (Fig. 2A). ADRP expression was also evaluated by Western blot, and the result was the same as that observed by immunofluorescence (Fig. 2B). Therefore, we concluded that Ang II could induce LD accumulation in podocytes.

### Effect of Ang II on the intracellular cholesterol content of podocytes

**Figure 3:**
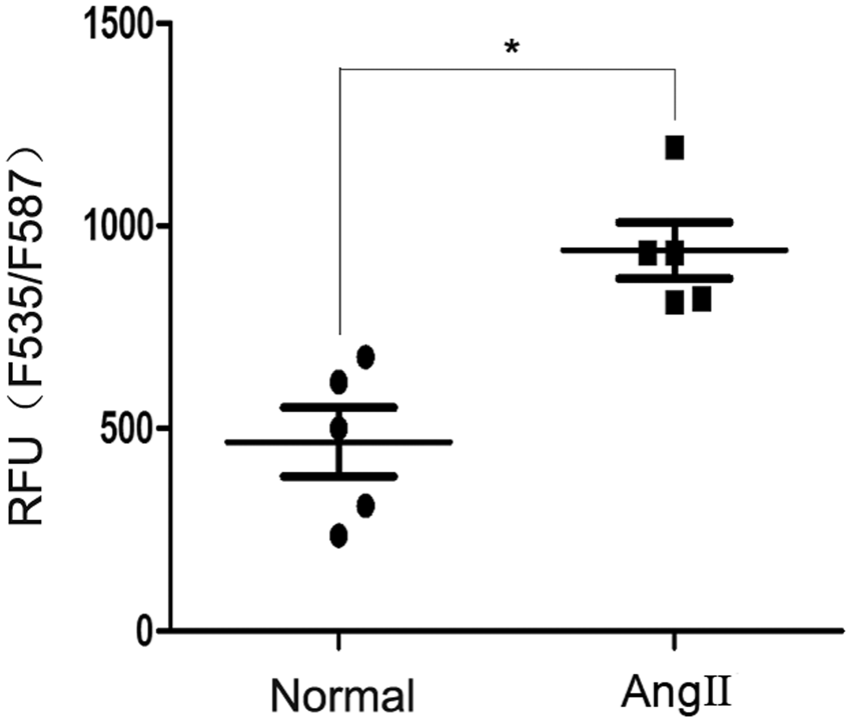
Effect of Ang II on the intracellular cholesterol content of podocytes. Podocytes were stimulated with Ang II (10^−7^ M) for 24 h. Cholesterol content was detected using a cholesterol quantitation kit. According to the kit, the cholesterol content is proportional to the fluorescence intensity. The figure shows the quantitative analysis of the fluorescence intensity in the two groups, n = 5, *p < 0.05 vs. the normal group.

Cholesterol homeostasis plays an important role in podocyte physiology^4^. As the exposure of podocytes in culture to Ang II caused LD accumulation and cholesterol is a major component of LD, we next tested the effect of Ang II on intracellular cholesterol. Cholesterol content was determined using a fluorometric assay according to the instructions of a cholesterol quantitation kit, in which cholesterol content was proportional to fluorescence intensity. The results showed that the fluorescence intensity in Ang II-treated podocytes was nearly two-fold higher than that in normal podocytes (Fig. 3). Thus, Ang II increased the cholesterol content of podocytes.

### Effects of Ang II on the mRNA and protein expression of molecules related to cholesterol metabolism in podocytes

**Figure 4:**
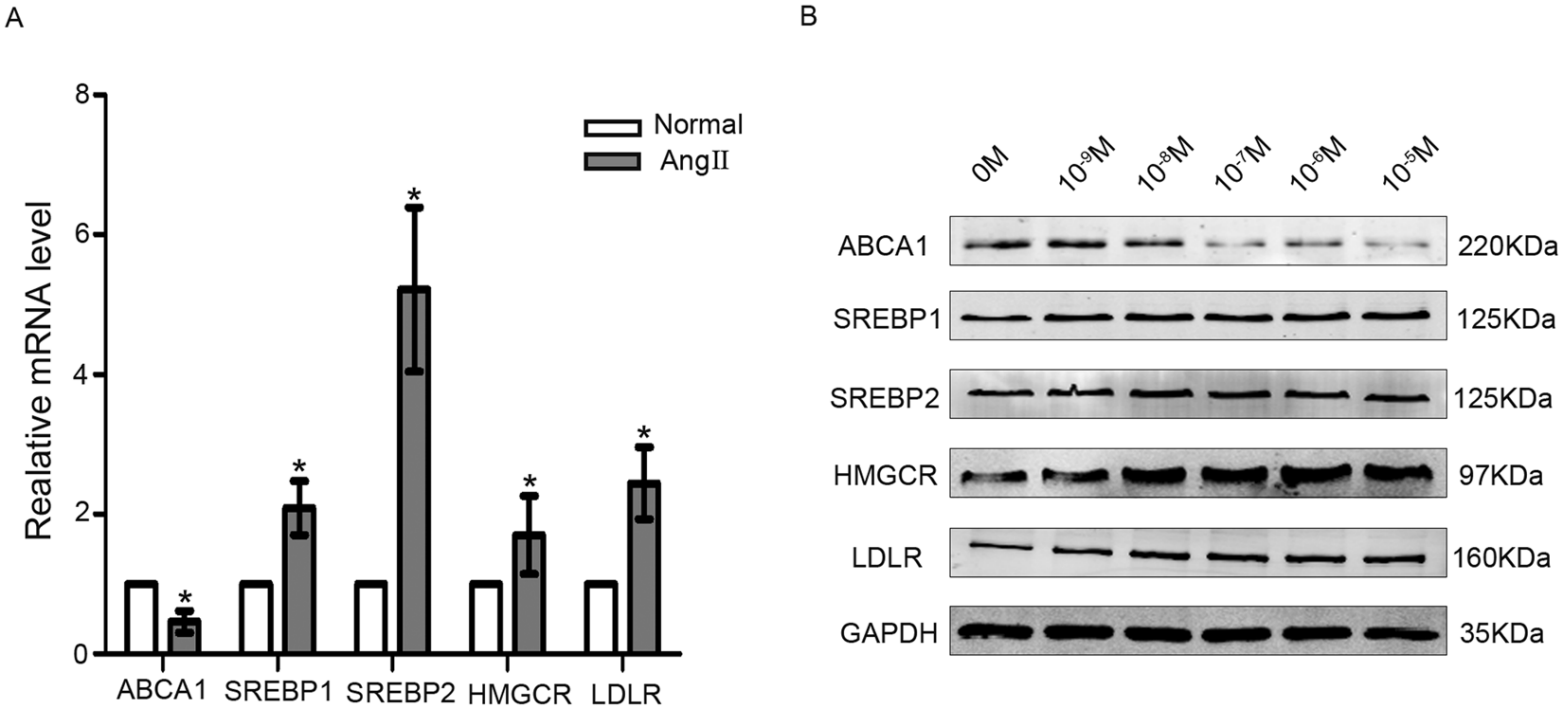
Effects of Ang II on the mRNA and protein expression of molecules related to cholesterol metabolism in podocytes. (**A**) Podocytes were treated with Ang II (10^−7^ M) for 24 h. The histogram shows the changes in the relative mRNA levels of the cholesterol metabolism-related molecules ABCA1, SREBP1, SREBP2, HMGCR and LDLR in the two groups, demonstrating that Ang II decreased the mRNA level of ABCA1 but significantly increased the mRNA levels of SREBP1, SREBP2, HMGCR and LDLR. n = 3, *p < 0.05 vs. the normal group. (**B**) Podocytes were treated with various concentrations of Ang II (10^−9^–10^−5^ M) for 24 h. Representative Western blots of ABCA1, SREBP1, SREBP2, HMGCR and LDLR, demonstrating that Ang II decreased the protein level of ABCA 1 but clearly increased the protein levels of SREBP1, SREBP2, HMGCR and LDLR. n = 3, *p < 0.05 vs. the normal group. For clarity lanes were cropped from different gels. Full-length blots are presented in Fig. S4.

We showed above that Ang II increased podocyte cholesterol content. The fine regulation of cholesterol homeostasis is maintained via different mechanisms^3^. We next analyzed the expression of several important molecules related to *de novo* cholesterol synthesis and cholesterol influx and efflux. The mRNA level of the cholesterol efflux-related molecule ABCA1 was lower in Ang II-treated podocytes than in normal podocytes, while the mRNA levels of the cholesterol uptake-related molecule LDLR and the cholesterol synthesis-related molecules SREBP1, SREBP2 and HMGCR were higher in Ang II-treated podocytes than in normal podocytes (Fig. 4A).

The protein levels of cholesterol metabolism-related molecules were assessed by Western blot. Podocytes were treated with Ang II at various concentrations (10^−9^–10^−5^ M) for 24 h. We found that Ang II exposure induced a decrease in ABCA 1 levels in a dose-dependent manner, whereas the expression levels of LDLR, SREBP1, SREBP2 and HMGCR were increased in a dose-dependent manner by Ang II (Fig. 4B). Quantitation of the Western blot results is shown in Fig. S1.

Thus, the results demonstrated that exposure of podocytes in culture to Ang II caused cholesterol accumulation in association with decreased cholesterol efflux, increased cholesterol uptake and increased cholesterol synthesis.

### CD ameliorated Ang II-induced podocyte cholesterol accumulation and injury

**Figure 5:**
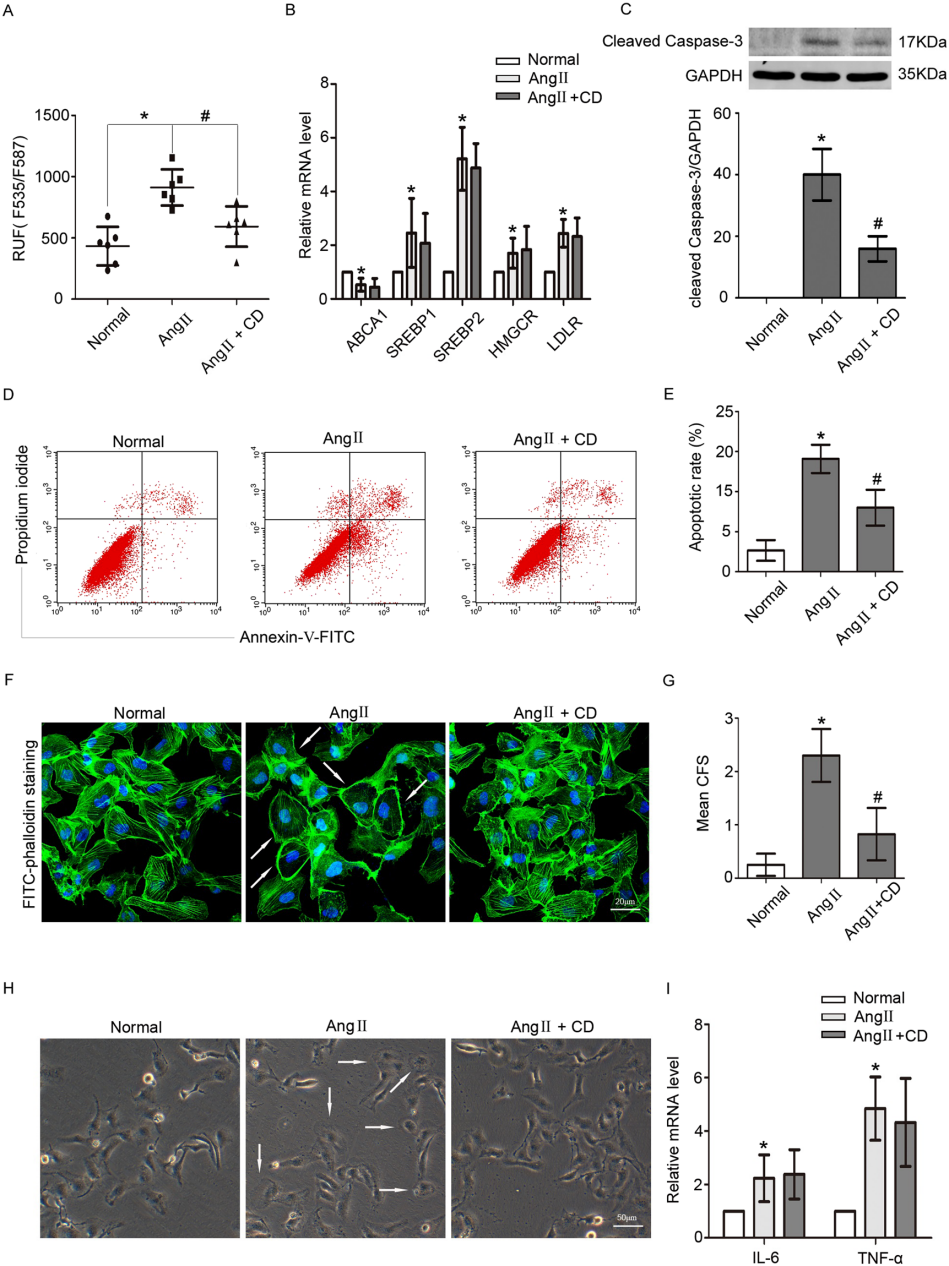
CD ameliorated Ang II-induced cholesterol accumulation and injury in podocytes. Podocytes were pretreated with CD and then stimulated with Ang II. (**A**) Quantitative analysis of the fluorescence intensity in different groups, indicating that CD reduced Ang II-induced cholesterol accumulation. n = 6, *p < 0.05 vs. the normal group, ^#^ *p* < 0.05 vs. the Ang II group. (**B**) The histogram shows the changes in the relative mRNA levels of the cholesterol metabolism-related factors ABCA1, SREBP1, SREBP2, HMGCR and LDLR in three different groups, demonstrating that CD did not affect the Ang II-mediated expression levels. n = 3, *p < 0.05 vs. the normal group. (**C**) Representative Western blot and quantitative analysis of Cleaved Caspase-3 in different groups. n = 3, *p < 0.05 vs. the normal group, ^#^ *p* < 0.05 vs. the Ang II group. For clarity lanes were cropped from the same gel. Full-length blots are presented in Fig. S5. (**D**) Flow cytometry analysis of podocyte apoptosis in different groups. (**E**) Quantitative analysis of the podocyte apoptotic rate in different groups, demonstrating that Ang II induced podocyte apoptosis, but CD pretreatment lowered the apoptosis rate. n = 3, *p < 0.05 vs. the normal group, ^#^ *p* < 0.05 vs. the Ang II group. (**F**) Representative FITC-phalloidin staining of the podocyte actin cytoskeleton in different groups (magnification ×400). Scale bar, 20 μm. (**G**) Quantitative analysis of CFS (n = 100). n = 3, *p < 0.05 vs. the normal group, ^#^ *p* < 0.05 vs. the Ang II group. Ang II induced podocyte cytoskeleton reorganization, which was improved by CD pretreatment. (**H**) Representative spreading images of podocytes in different groups (original magnification ×200). Scale bar, 50 μm. Ang II inhibited podocyte spreading, but CD pretreatment promoted podocyte spreading. (**I**) The histogram shows the changes in the relative mRNA levels of IL-6 and TNF α. n = 3, *p < 0.05 vs. the normal group.

Ang II is a risk factor for podocyte damage^13, 14^. Whether Ang II-induced cholesterol accumulation causes podocyte injury is unclear. We next assessed the relationship between Ang II-induced cholesterol deposition and podocyte injury by pretreating Ang II-treated podocytes with CD. The data showed that CD decreased Ang II-induced cholesterol accumulation by nearly one-half without influencing the mRNA expression of cholesterol metabolism-related molecules (Fig. 5A and B); CD decreased Ang II-induced upregulation of Cleaved Caspase-3 and Ang II-induced podocyte apoptosis (Fig. 5C–E).

Podocyte cytoskeleton is important for various podocyte functions, such as migration and spreading. Reorganization of the podocyte cytoskeleton could promote cell detachment and apoptosis^15^. Thus we next examined the cytoskeleton in podocytes. The stress fibers in normal podocytes became disorganized after Ang II treatment, exhibiting peripheral actin bundles and scattered actin fragments. However, the cytoskeleton disruption was partially improved by CD (Fig. 5F and G). Ang II also evidently inhibited podocyte spreading, as the podocytes lost foot process and became round, and the inhibition of cell spreading was partially rescued by CD (Fig. 5H). In addition, we examined the expression of podocin, which is vital for podocyte function. We found that CD-induced cholesterol efflux upregulated podocin expression (Fig. S2). Ang II is a pro-inflammatory factor. To exclude the effect of inflammation due to Ang II, we analyzed the expression of the inflammatory factors IL-6 and TNF α and found that both were upregulated by Ang II but not affected by CD (Fig. 5I).

Thus, our results indicated that the cholesterol overload was associated with podocyte injury and that cholesterol reduction could ameliorate the damage to podocytes.

### Simvastatin did not improve Ang II-induced podocyte cholesterol accumulation and apoptosis

**Figure 6:**
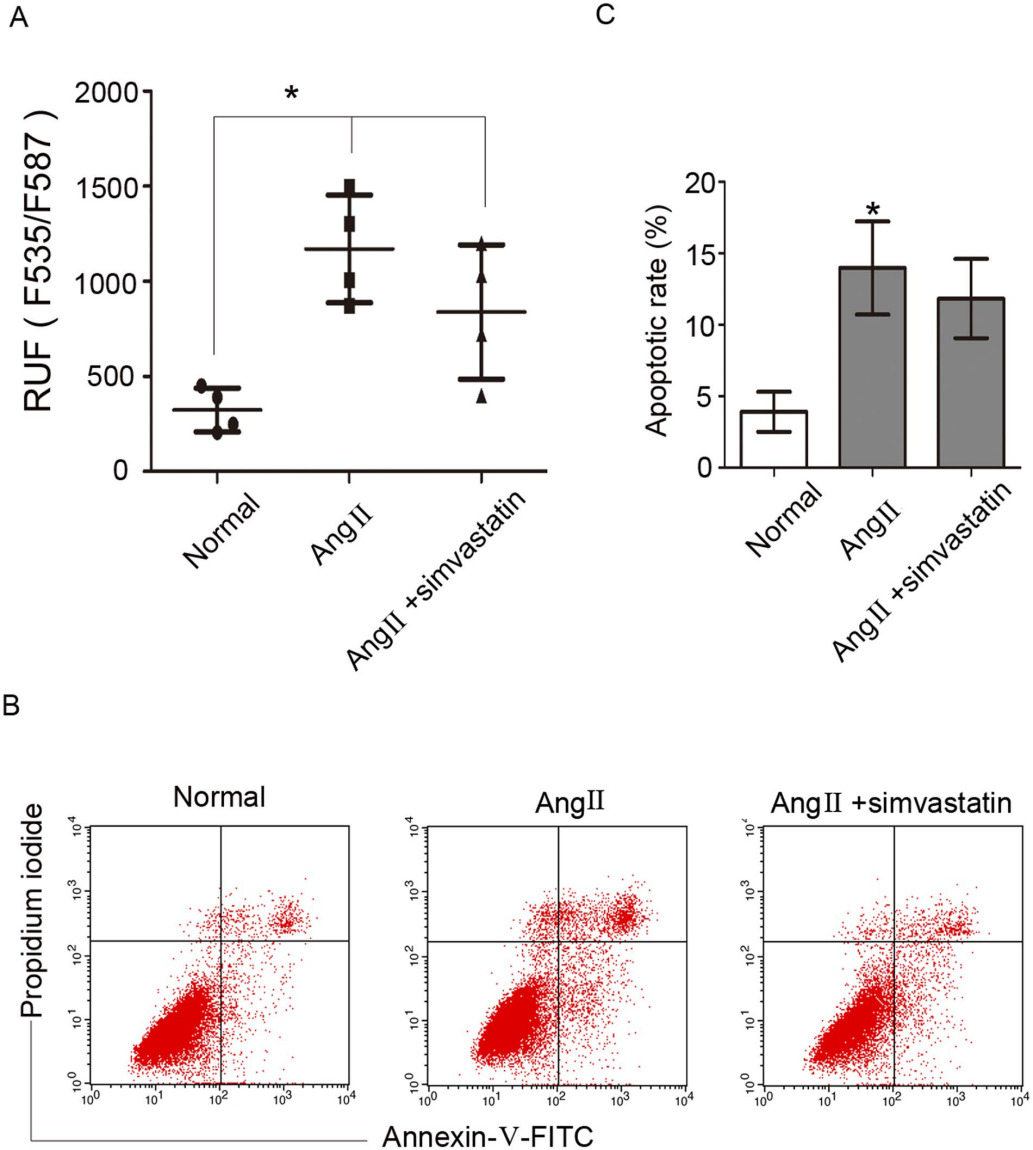
Simvastatin did not improve Ang II-induced podocyte cholesterol accumulation and apoptosis. Podocytes were pretreated with simvastatin and stimulated with Ang II. (**A**) Quantitative analysis of the fluorescence intensity in different groups, demonstrating that there was no difference in fluorescence intensity between the Ang II + simvastatin group and the Ang II group. n = 4, *p < 0.05 vs. normal group. (**B**,**C**) Flow cytometry analysis of podocyte apoptosis in different groups, demonstrating that simvastatin pretreatment did not ameliorate the apoptosis induced by Ang II. n = 3, *p < 0.05 vs. the normal group.

Many studies have reported that statins can decrease the mortality rate of cardiac events^16^. Statins are HMGCR antagonists that can inhibit cholesterol synthesis. We next examined the effect of statins on podocyte cholesterol metabolism by treating podocytes with simvastatin. Our data demonstrated that simvastatin did not reduce Ang II-induced cholesterol accumulation (Fig. 6A). Flow cytometry results showed that simvastatin did not decrease Ang II-induced podocyte apoptosis (Fig. 6B and C).

## Discussion

Experimental studies have shown that glomerular capillary hypertension and impaired filtration function with subsequent protein overload play a pathogenic role in the progression of CKD; RAS inhibition can reduce proteinuria more effectively than non-RAS inhibition therapy when comparable BP control is provided^17, 18^. Ang II, a mediator of RAS, has emerged as a multifunctional cytokine with growth factor, pro-fibrogenic and pro-inflammatory properties^11, 19, 20^. Ang II also plays a role in lipid metabolism. Ma *et al*. reported that Ang II induced lipid accumulation in human renal mesangial cells (HMCs)^21^. Borghi *et al*. reported that Ang II influenced the cholesterol level in macrophages and foam cells^22^. The present study showed that Ang II induced LD deposition in podocytes, as indicated by Oil Red O and Nile Red staining and ADRP expression, and losartan could reduce the LD accumulation induced by Ang II. Therefore, we hypothesized that Ang II affected podocyte lipid metabolism, and that an elevated Ang II level could lead to LD accumulation in podocytes.

LDs are organelles that store triacylglycerides and cholesterol esters^23^. Cholesterol esters in LDs are maintained in a balance with free cholesterol in the cytoplasm^24, 25^. Cholesterol plays an important role in podocytes^26^. Huber *et al*. and Schermer *et al*. reported that cholesterol was essential for podocin regulation of the transient receptor potential C channel protein (TRPC 6)^27, 28^. However, excess cholesterol is harmful to podocyte function. Pedigo *et al*. reported that local TNF could cause cholesterol-dependent apoptosis in podocytes by reducing ABCA1-mediated cholesterol efflux and reducing cholesterol esterification by sterol-O-acyltransferase 1 (SOAT1)^29^. Sorensson *et al*. reported that cholesterol overload negatively affected the binding of podocyte SD proteins to each other^30^. Our data showed that Ang II increased the total cholesterol content nearly twofold compared with normal podocytes, thus leading to podocyte cholesterol overload. Evaluation of the mRNA and protein levels of cholesterol metabolism-related molecules demonstrated that ABCA1 expression was downregulated by Ang II, whereas the expression of SREBP1, SREBP2, HMGCR, and LDLR was upregulated by Ang II; these findings indicated that Ang II increased cholesterol synthesis and influx and decreased cholesterol efflux. Therefore, we speculated that Ang II disrupted cholesterol metabolism by affecting the three cholesterol metabolism pathways.

Previous studies have shown that Ang II can induce podocyte injury, but the precise mechanism was unknown. The present study demonstrated that Ang II induced podocyte injury by affecting cholesterol metabolism. We used the well-known cholesterol efflux inducer CD to decrease the cholesterol level induced by Ang II, and discovered that CD prevented podocyte apoptosis and maintained the podocyte cytoskeleton and spreading ability. We also evaluated the expression of podocin and found that CD improved Ang II-induced podocin downregulation. Though previous studies have reported that cholesterol is required for podocin function but not for its expression^28^, we speculated that well-balanced cholesterol may have a supplementary function in maintaining podocin expression. Ang II caused podocyte injury directly via multiple pathways, such as inflammation. To differentiate the direct effects of Ang II from cholesterol-induced injury we analyzed the expression of the inflammatory factors IL-6 and TNF α and found that both were upregulated by Ang II but not affected by CD. Thus, our study demonstrated that Ang II-induced podocyte injury was partially cholesterol-dependent.

However, we found that the HMGCR antagonist simvastatin did not reduce Ang II-induced cholesterol accumulation and podocyte apoptosis. As cholesterol homeostasis is regulated by multiple mechanisms, we speculated that simvastatin alone might play a small role. Merscher *et al*. reported that increased cholesterol accumulation was associated with ABCA1 downregulation in podocytes exposed to sera from patients with DKD^5^. Pedigo *et al*. reported that local TNF caused cholesterol-dependent apoptosis in podocytes by reducing ABCA1-mediated cholesterol efflux^29^. Thus, we hypothesized that abnormal cholesterol efflux may play a major role in cholesterol accumulation mediated by Ang II in podocytes. ABCA1 and other cholesterol efflux-related molecules should be considered in Ang II-induced cholesterol accumulation. In addition, our data showed that CD only partially ameliorated Ang II-induced injury. Thus, we speculated that another mechanism must participate in podocyte injury mediated by Ang II.

In summary, the present study demonstrated that Ang II induced cholesterol accumulation. Ang II-induced podocyte injury was partially cholesterol-dependent. The association between Ang II and cholesterol metabolism may provide a new understanding of RAS-associated podocyte injury and CKD progression.

## Methods

### Cell culture

Conditionally immortalized human podocytes were kindly provided by Dr. Moin A. Saleem (Academic Renal Unit, Southmead Hospital, Bristol, UK) and cultured in RPMI 1640 medium (HyClone, USA) containing 10% fetal bovine serum (FBS) (Gibco, USA), 100 U/mL penicillin G, 100 μg/mL streptomycin, 1 × insulin, transferrin, and selenium (ITS) (Invitrogen, USA) in a 33 °C incubator for proliferation. When the podocytes reached approximately 80% confluence, the cells were subcultured in a 37 °C incubator with ITS-free medium for 7–14 days for differentiation. Differentiated podocytes were serum starved in RPMI 1640 medium without FBS for all experiments. Then, the cells were stimulated with 10^−9^–10^−5^ M Ang II (Enzo Life Science, Netherlands) for 24 h. For losartan treatment, serum-starved podocytes were treated with 10^−5^ M losartan for 24 h. For CD experiments, serum-starved podocytes were pretreated with 5 mM methyl-β-cyclodextrin (Sigma-Aldrich, USA) for 1 h. For statin experiments, serum-starved podocytes were pretreated for 1 h with 1 μM simvastatin (Sigma-Aldrich, USA).

### Oil Red O staining

Cell-climbing films (cells growing on a glass slide) were fixed with 4% paraformaldehyde for 30 min at 4 °C and rinsed with distilled water. Then the films were rinsed with 60% isopropanol for 1 min. The films were incubated with Oil Red O (Sigma-Aldrich, USA) working solution for 15 min at room temperature, rinsed again for 1 min with 60% isopropanol and then returned to distilled water. Finally, the films were counterstained with hematoxylin for 1 min. All microscopy images were recorded using a 12.8-megapixel camera (DP72, Olympus, Japan).

### Nile Red staining

Cell-climbing films were rinsed with PBS and stained with Nile Red (Sigma-Aldrich, USA) working solution for 10 min at room temperature. Afterwards, the films were rinsed with PBS. All microscopy images were recorded using a 12.8-megapixel camera (DP72, Olympus, Japan).

### Immunofluorescence assay

The cell-climbing films were fixed with 4% paraformaldehyde for 30 min at 4 °C and incubated first with a guinea pig anti-ADRP antibody (1:100, PROGEN Biotechnik, Germany) overnight at 4 °C, and then with tetramethylrhodamine (TRITC)-conjugated goat anti-guinea pig immunoglobulin G (IgG) (1:100, Thermo Scientific, USA) at 37 °C for 60 min in the dark. The nuclei were counterstained with 4′, 6-diamidino-2-phenylindole (DAPI) (Antgene, China) for 5 min. All microscopy images were recorded using a confocal microscope (Olympus, Japan).

### Cholesterol quantification

A cholesterol quantitation kit (Sigma-Aldrich, USA) was used to determine the cholesterol content of podocytes. Cells (1 × 10^6^) were extracted with 200 μL of chloroform:isopropanol:IGEPAL CA-630 (7:11:0.1) in a microhomogenizer. The samples were centrifuged at 13000 *g* for 10 min to remove insoluble material. Next, the samples were transferred in the organic phase to a new tube and air dried at 50 °C to remove any residual organic solvent. Dried lipids were dissolved in 200 μL of the cholesterol assay buffer and then vortexed until the mixture was homogenous. The reaction mixtures were prepared according to the kit instruction, and the cholesterol content of the lipid solution was determined using a fluorometric assay. The fluorescence intensity was analyzed in a fluorescence microplate reader with an excitation wavelength of 535 nm and an emission wavelength of 587 nm.

### Real-time PCR

Total RNA was extracted from cells using TRIzol reagent (Invitrogen, USA), and the concentration of the collected RNA was determined by spectrophotometry. Next, cDNA was synthesized and analyzed using a real-time fluoresce-based quantitative PCR machine (Illumina Eco, USA). The specificity of real-time PCR was determined using melting curve analysis. GAPDH was used as an internal standard. The primers used in this study were in Table 1.

**Table 1:** Primers used in Real-time PCR.

Gene	Sense	Antisense
AT1	CTTTGCCACTATGGGCTGTCTA	GATGCAGGTGACTTTGGCTACA
AT2	CTGGCTCTTTGGACCTGTGAT	CTGACCATTGGGCATATTTCTC
ABCA1	CCTTGGGTTCAGGGGATTAT	TCATGCTGGTGTCTTTCTGG
SREBP1	GAGCCATGGATTGCACTTTC	CAGGAAGGCTTCAAGAGAGG
SREBP2	GCCTCAACCTCAAACTCAGC	TACTGTCTGCACCTGCTGCT
HMGCR	TCCCTGGGAAGTCATAGTGG	AGGATGGCTATGCATCGTGT
LDLR	TGTTCCAAGGGGACAGTAGC	TGCAGTTTCCATCAGAGCAC
Podocin	TCCGCACAAGGAGAACAAGAG	TGATGAAGAGCAGGGAAATGAG
IL-6	GGTCCAGTTGCCTTCTCCC	GTGCCTCTTTGCTGCTTTC
TNFα	TCAGAGGGCCTGTACCTCAT	GGAAGACCCCTCCCAGATAG
GAPDH	CGGAGTCAACGGATTTGGTCGTAT	AGCCTTCTCCATGGTGGTGAAGAC

### Western blot

Total protein was extracted from cells using radio immunoprecipitation assay (RIPA) (Beyotime, China) buffer containing protease/phosphatase inhibitors (Sigma-Aldrich, USA) and centrifuged at 13,000 g and 4 °C for 5 min. The supernatants were mixed with loading buffer and boiled at 100 °C for 5 min. The protein concentration was measured using a BCA protein assay (Thermo Scientific, USA). Equal amounts of protein were separated using 10% sodium dodecyl sulfate-polyacrylamide gel electrophoresis (SDS-PAGE) and then transferred to polyvinylidene fluoride (PVDF) membranes (Millipore, USA). The membranes were incubated overnight at 4 °C with a primary antibody (ABCA1 mouse monoclonal antibody, 1:1000, Novus Biologicals, USA; SREBP1 mouse monoclonal antibody, 1:500, Novus Biologicals, USA; SREBP2 mouse monoclonal antibody, 1:200, Santa Cruz, USA; HMGCR rabbit monoclonal antibody, 1:500, Abcam, UK; LDLR rabbit polyclonal antibody, 1:1000, Novus Biologicals, USA; ADRP guinea pig polyclonal antibody, 1:2000, PROGEN Biotechnik, Germany; Cleaved Caspase-3 rabbit monoclonal antibody, 1:2000, CST, USA; and GAPDH mouse monoclonal antibody, 1:2000, Antgene, China). An Alexa Fluor 680/790-labeled goat anti-rabbit/goat anti-mouse/goat anti-guinea pig IgG antibody (1:25,000, LI-COR Biosciences, USA) was used as the secondary antibody, and the blots were visualized using an Odyssey infrared imaging system (LI-COR Biotechnology, USA).

### Apoptosis assay

The expression of Cleaved Caspase-3, as determined by Western blot, was used to evaluate podocyte apoptosis. Flow cytometry was also performed to assess the degree of apoptosis in podocytes. Annexin-V-FITC and PI (Bio Legend, USA) were used to identify apoptotic cells according to the manufacturer’s instructions. Cells in the upper-right and lower-right quadrants were classified as apoptotic.

### Staining of the actin cytoskeleton

Podocytes were fixed with 4% paraformaldehyde at 4 °C for 30 min, washed with PBS 3 times for 5 min each, and stained with 2.5 μg/mL FITC-phalloidin (Sigma-Aldrich, USA) overnight at room temperature. The nuclei were counterstained with DAPI for 5 min. All microscopy images were recorded using a confocal microscope (Olympus, Japan).

The cortical F-actin score (CFS) was assessed to quantify the degree of cytoskeletal reorganization^15^. Specifically, the cytoskeletal reorganization for each cell was scored on a scale ranging from 0 to 3 based on the degree of cortical F-actin ring formation (score = 0, no cortical F-actin, normal stress fibers; score = 1, cortical F-actin deposits below ½ of the cell border; score = 2, cortical F-actin deposits exceeding ½ of the cell border; score = 3, complete cortical ring formation and/or total absence of central stress fibers).

### Cell spreading assay

Podocytes were seeded into a 6-well plate. The morphology of the podocytes was observed under an inverted phase-contrast microscope. Spreading cells had extended processes, whereas non-spreading cells were round.

### Statistical analyses

All experiments were performed at least three times, with similar results obtained between experiments. All values are presented as the mean with the SD and were analyzed using SPSS 17.0. Differences in the mean values were tested using Student’s t test or one-way ANOVA. Differences were considered statistically significant at p < 0.05.

### Data availability

The datasets generated and/or analyzed during the current study are available from the corresponding author on reasonable request.

## Electronic supplementary material

supplementary information
