# Supplementary material for: Angiotensin II induces cholesterol accumulation and injury in podocytes
Source: Sci Rep. 2017 Sep 6;7:10672. doi: 10.1038/s41598-017-09733-w (PMC5587570; doi:10.1038/s41598-017-09733-w)
Supplement: Supplementary file 1 — supplementary information [file 41598_2017_9733_MOESM1_ESM.pdf]

Angiotensin Ⅱ induces cholesterol accumulation and injury in podocytes

Yingjie Yang, Qian Yang, Jian Yang, Yiqiong Ma & Guohua Ding*

Division of Nephrology, Renmin Hospital of Wuhan University, Wuhan, Hubei 430060, China

Correspondence and requests for materials should be addressed to Guohua Ding (ghxding@gmail.com)

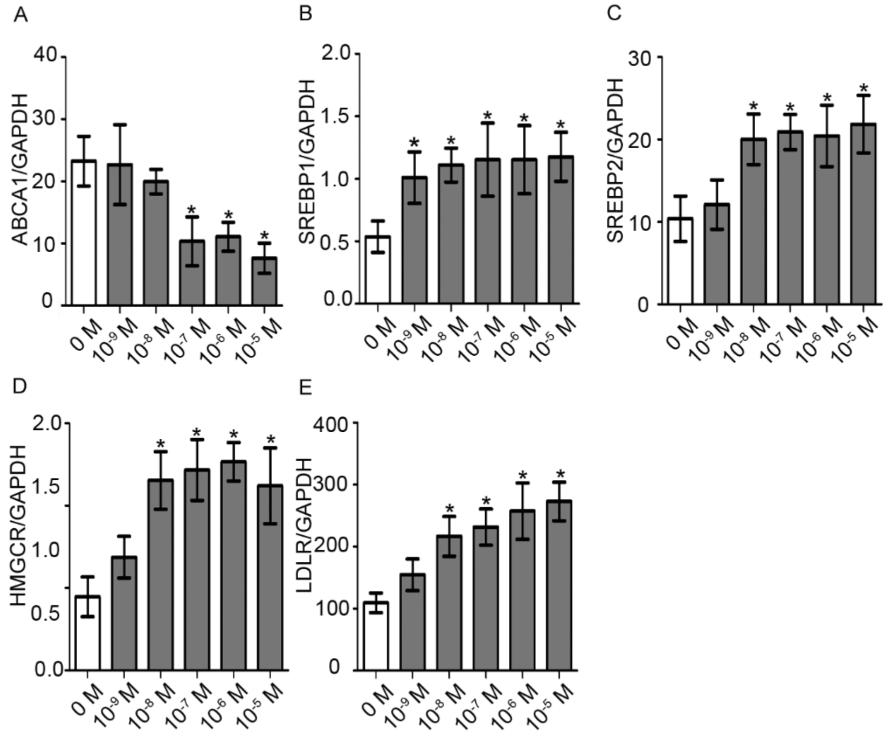

Figure S1. Quantitation of the Western blots of cholesterol metabolism-related molecules.

Podocytes were treated with various concentrations of AngII (10^{-9}-10^{-5}M) for 24 h. The histogram above represents the quantitation of the Western blots of cholesterol metabolism-related molecules. n = 3, *p < 0.05 vs. the normal group. AngII decreased the protein level of ABCA1 but clearly increased the protein levels of SREBP1, SREBP2, HMGCR and LDLR.

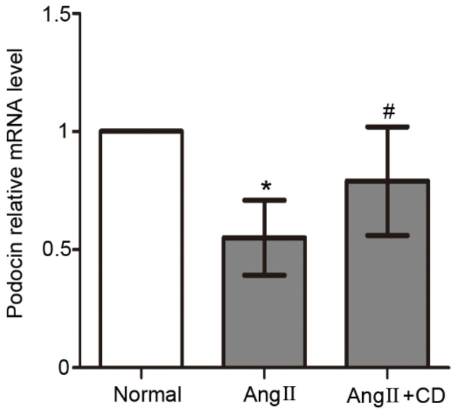

Figure S2. The effect of CD on podocin expression in podocytes.

Podocytes were pretreated with CD and then stimulated with Ang Ⅱ. The graph shows quantitative analysis of the mRNA level of podocin. n = 3, *p < 0.05 vs. the normal group. #p < 0.05 vs. the Ang Ⅱ group.

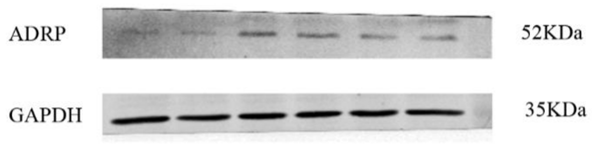

Figure S3. Full-length blots of Figure 2B.

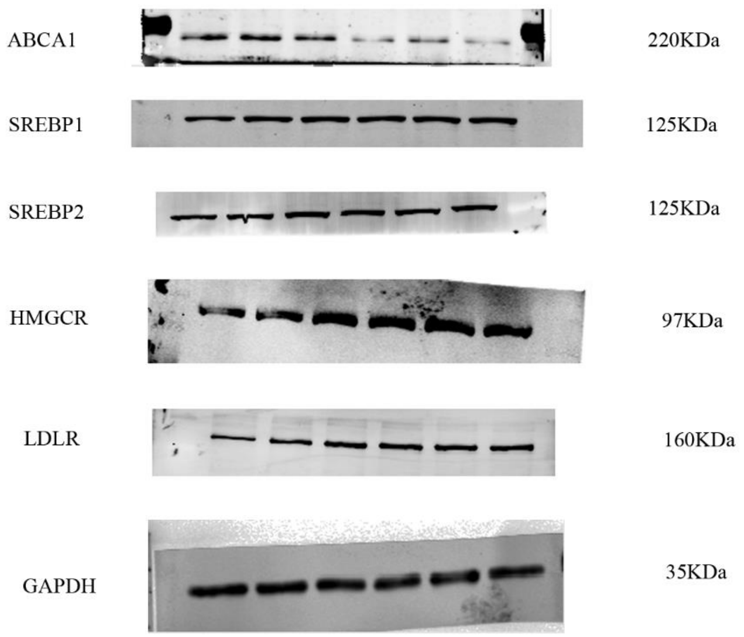

Figure S4. Full-length blots of Figure 4B.

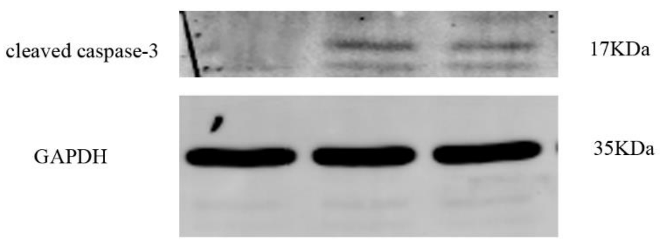

Figure S5. Full-length blots of Figure 5C.

Supplementary Table 1. Specificity and reactivity of antibodies used in the manuscript.

**Antibody**		**Reactivity**
ADRP	guinea pig polyclonal antibody	Human, mouse, rat, dog, bovine
ABCA1	Mouse monoclonal antibody	Human, mouse, rat, rabbit
SREBP1	Mouse monoclonal antibody	Human, mouse, rat, canine, chicken, hamster, golden syrian hamster
SREBP2	Mouse monoclonal antibody	Mouse, rat, human
HMGCR	Rabbit monoclonal antibody	Mouse, rat, human
LDLR	Rabbit polyclonal antibody	Human, mouse, primate
Cleaved caspase-3	Rabbit monoclonal antibody	Human
GAPDH	Mouse monoclonal antibody	Human, mouse, rat, rabbit
